# Catalytic strategies of the non-heme iron dependent oxygenases and their roles in plant biology

**DOI:** 10.1016/j.cbpa.2016.02.017

**Published:** 2016-04

**Authors:** Mark D White, Emily Flashman

**Affiliations:** Chemistry Research Laboratory, 12 Mansfield Road, Oxford OX1 3TA, UK

## Abstract

Non-heme iron-dependent oxygenases catalyse the incorporation of O_2_ into a wide range of biological molecules and use diverse strategies to activate their substrates. Recent kinetic studies, including *in crystallo*, have provided experimental support for some of the intermediates used by different subclasses of this enzyme family. Plant non-heme iron-dependent oxygenases have diverse and important biological roles, including in growth signalling, stress responses and secondary metabolism. Recently identified roles include in strigolactone biosynthesis, O-demethylation in morphine biosynthesis and regulating the stability of hypoxia-responsive transcription factors. We discuss current structural and mechanistic understanding of plant non-heme iron oxygenases, and how their chemical/genetic manipulation could have agricultural benefit, for example, for improved yield, stress tolerance or herbicide development.

## Highlights

• Current evidence for iron-oxo reactive intermediates is reviewed.
• *In crystallo* intermediates detected in a native extradiol dioxygenase reaction.
• Carotenoid cleavage dioxygenases catalyse strigolactone biosynthesis.
• Identification of plant cysteine oxidases involved in the plant hypoxic response.
• Applications of enzyme manipulation to plant biology and agriculture are discussed.

## Graphical abstract

**Figure:**
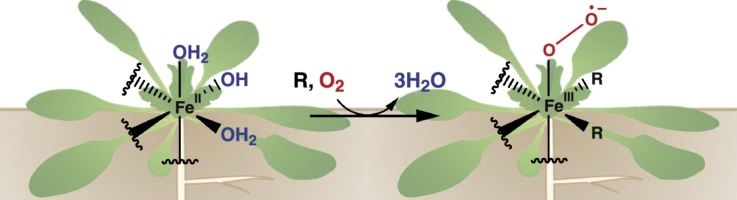

**Current Opinion in Chemical Biology** 2016, **31**:126–135

This review comes from a themed issue on **Bioinorganic chemistry**

Edited by **R David Britt** and **Emma Raven**

For a complete overview see the Issue and the Editorial

Available online 23rd March 2016

**http://dx.doi.org/10.1016/j.cbpa.2016.02.017**

1367-5931/© 2016 The Authors. Published by Elsevier Ltd. This is an open access article under the CC BY license (http://creativecommons.org/licenses/by/4.0/).

## Introduction

Non-heme iron-dependent oxygenases are a widespread family of functionally diverse enzymes that facilitate the incorporation of molecular oxygen into a range of biomolecules, reactions that are often inaccessible through synthetic approaches. Motivated by biomimetic catalyst design, extensive research has been conducted to understand their chemistry, providing detailed mechanistic insights into a number of associated enzyme subfamilies. Unique or unifying catalytic strategies employed by these enzymes continue to be identified, as do novel and important biological roles. In this Review, we briefly highlight recent advances in the mechanistic understanding of non-heme (mono-) iron dependent oxygenases before focussing on the (ever-emerging) range of important roles that these enzymes play in plant biology. Targeted manipulation of these enzymes could have beneficial effects for plant growth and/or stress tolerance, addressing the 21st century issue of food security. We therefore highlight those of potential agricultural (and/or health) interest.

## Resolving intermediates in non-heme iron dependent oxygenase catalysis

**Figure 1:**
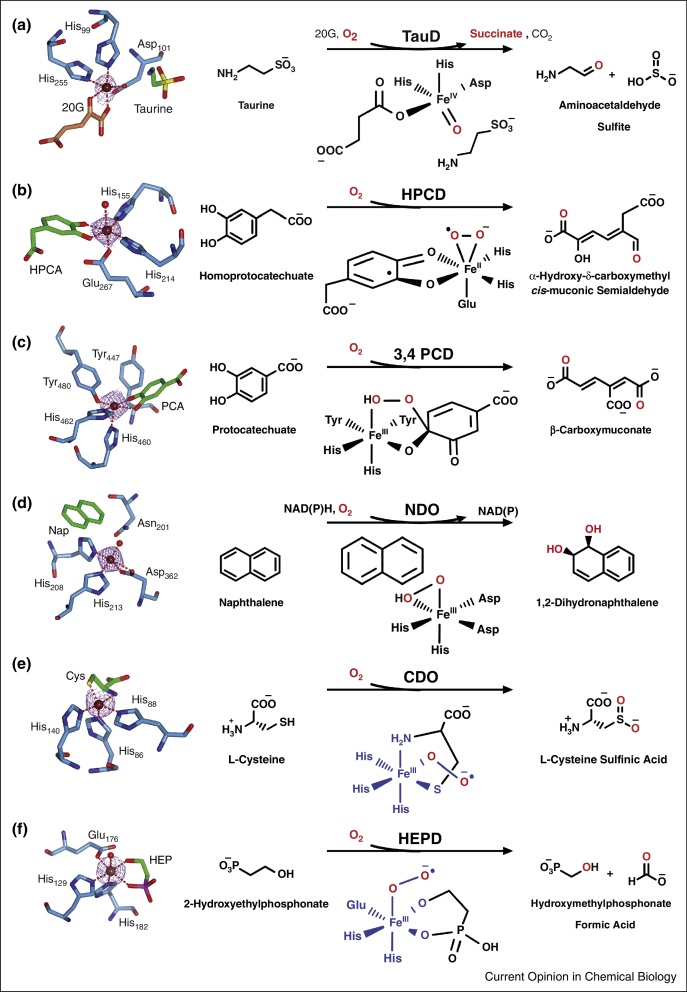
Non-heme iron-dependent oxygenase-catalysed reactions, active site structures and reaction intermediates. **Left panels**: The active site structures of non-heme iron-dependent oxygenases, showing the coordination of amino acid residues (light blue cylinders), substrate (green cylinders) and co-substrate (coral cylinders) to the iron (maroon sphere). A purple mesh shows the electron density of the metal cofactor and water molecules are represented as red balls. **Right panels**: The corresponding non-heme iron-dependent oxygenase reaction schemes and iron-oxo intermediates, following the route of molecular oxygen (red). Unresolved/currently proposed reaction species are coloured blue. For references, see text. **(a)** Taurine Dioxygenase (TauD) catalyses oxidation of taurine to aminoacetaldehyde and sulphite via a high valent Fe(IV)-oxo species (PDB ID: 1GQW). **(b)** Homoprotocatechuate dioxygenase (HPCD) catalyses the oxidative cleavage of homoprotocatechuate (HPCA) to α-hydroxy-δ-carboxymethyl-*cis*-muconic-semialdehyde using a Fe(II)-superoxo/semiquinone intermediate (PDB ID: 4GHG). **(c)** Protocatechuate 3,4-Dioxygenase (3,4-PCD) catalyses the oxidative cleavage of protocatechuate (PCA) to β-carboxymuconate via a Fe(III)-alkylperoxo intermediate. (PDB ID: 3PCA). **(d)** Naphthalene Dioxygenase (NDO) catalyses the dihydroxylation of naphthalene (Nap) via a Fe(III)-(hydro)peroxo intermediate (PDB ID: 1O7G). **(e)** Cysteine dioxygenases (CDO) catalyse the oxidation of cysteine (Cys) to cysteine sulfinic acid via a (putative) Fe(III)-superoxo intermediate (PDB ID: 2IC1). **(f)** 2-Hydroxyethylphosphonate dioxygenase (HEPD) catalyse the oxidation of 2-hydroxyethylphosphonate (HEP) to hydroxymethylphosphonate and formic acid via a (putative) Fe(III)-superoxo species (PDB ID: 3GBF).

To activate O_2_, non-heme iron-dependent oxygenases use a redox active iron, coordinated octahedrally at their active site with water, usually in a vicinal facial triad arrangement [1]. During turnover, H_2_O is sequentially displaced by substrate/co-substrate and O_2_, which is reductively activated enabling a specific substrate modification. Enzyme subfamilies are proposed to employ different reactive iron-oxo species to activate their substrate. These species are short-lived intermediates that are difficult to study, but methodological advances over the past 15 years have enabled an accumulation of indirect and direct evidence for their formation. For example, a combination of freeze trapping techniques and Mössbauer spectroscopy allowed the characterisation of a transient Fe(IV)-oxo species in 2-oxoglutarate (2OG)-dependent and pterin-dependent oxygenases (e.g. TauD and Phenylalanine hydroxylase, respectively, Figure 1a) [2, 3, 4]. This high valent intermediate has not been identified in all non-heme iron-dependent oxygenases (formation of an Fe(IV)-oxo intermediate usually requires co-oxidation of another species, e.g. 2OG) and others are proposed to modify their substrates via lower valent alternatives. Here we summarise current evidence for activating intermediates across other members of this enzyme family.

### Catechol dioxygenases

Catechol (intradiol and extradiol) dioxygenases catalyse the oxidative cleavage of catechol substrates as part of bacterial aromatic degradation pathways [5]. Extradiol dioxygenases cleave the C—C bond adjacent to a hydroxyl group to form ring-opened products where O atoms are incorporated into aldehyde and carboxylate functional groups (Figure 1b). Fe(II) is ligated by the common His/His/Glu motif [1] with substrate hydroxyl groups coordinating directly to the metal prior to O_2_ binding. Mechanistic hypotheses based on studies with homoprotocatechuate dioxygenase (HPCD) propose no overall change in the oxidation state of the Fe(II) during turnover of the wild type enzyme, with Fe(II) acting as a conduit for electron transfer between O_2_ and substrate to generate Fe(II)-superoxo and semiquinone radical intermediates (Figure 1b) which recombine to form an alkylperoxo intermediate [6]. These species have been observed *in crystallo* upon reaction of an anaerobic HPCD·Fe(II)·4-NC complex with O_2_, where 4-NC (4-nitrocatechol) is a non-physiological substrate used to slow the reaction (note care should be taken in interpreting the radical nature or oxidation status of intermediates observed crystallographically without additional analysis) [7]. The use of HPCD variants and substituted active site metals has allowed spectroscopic detection of catalytically competent Fe/Co/Mn(III)-superoxo intermediates upon slower rates of catalysis (reviewed in [6]). As only Fe(II) species have been observed in the native reaction, the Fe(II) state may indeed be maintained or it may be that an Fe(III)-superoxo is so transient that it remains undetectable with current technical capabilities. Recently, *in crystallo* studies of homogentisate 1,2-dioxygenase (HGDO), an extradiol dioxygenase which catalyses conversion of homogentisate (HG) to maleylacetoacetate, revealed the equivalent intermediates to those seen *in crystallo* for HPCD, but in the reaction with native HG substrate. Exposing anaerobically prepared HGDO·Fe(II)·HG co-crystals to air followed by flash-cooling allowed observation of Fe(II)-superoxo·semiquinone radical, alkylperoxo and product bound intermediates [8^•^], supporting the proposed mechanism for this class of enzyme (though again, it may be that *in crystallo* studies cannot detect very transient higher valent intermediates).

Intradiol dioxygenases cleave the C—C bond of catechol enediol units, and use 2 tyrosine and 2 histidine residues to bind Fe(III). Fe(III) is unlikely to bind and activate O_2_, presenting a mechanistic conundrum. It is proposed, based predominantly on studies with protocatechuate 3,4-dioxygenase (3,4-PCD), that catechol substrates bind to the Fe(III) via both hydroxyls. Concerted O_2_ addition forms a Fe(III)-alkylperoxo species, before peroxo protonation results in O—O scission and a Criegee rearrangement to create an anhydride in the substrate ring which is attacked by the Fe(III)-hydroxide promoting cleavage [5]. None of these intermediates have been spectroscopically characterised, but *in crystallo* kinetic experiments using a slow-reacting substrate analogue (4-fluorocatechol) revealed species analogous with both proposed alkylperoxo (Figure 1c) and anhydride intermediates [9].

### Rieske dioxygenases

Rieske dioxygenases catalyse a diverse range of reactions, predominantly on arene substrates [10], a well-studied example being naphthalene dioxygenase (Figure 1d). Electrons for O_2_ reduction are supplied by NAD(P)H via a 2Fe-2S (Rieske) cluster. The proposed mechanism invokes O_2_ binding to an enzyme·substrate complex followed by electron transfer from the Rieske cluster to form an Fe(III) hydroperoxo intermediate (observed *in crystallo* for naphthalene dioxygenase [11]). The subsequent steps have not been resolved, but have been proposed to occur either via coupled O—O cleavage and substrate oxidation or via rearrangement of the hydroperoxo intermediate to cleave O—O and yield a high valent Fe(V)oxo-hydroxo species which subsequently oxidises the substrate (discussed in detail in [10]). Interestingly, a recent study correlating rates of Rieske cluster oxidation and product formation in benzoate 1, 2-dioxygenase implicates another possible mechanism, whereby substrate activation is achieved by a Fe(III)-superoxo species prior to electron transfer from the Rieske cluster [12]. It is possible that different oxidative approaches are utilised by different members of this diverse enzyme sub-class.

### Cysteine dioxygenases

Cysteine dioxygenases (CDOs) catalyse l-cysteine oxidation to cysteine sulfinic acid as part of taurine biosynthesis (Figure 1e) [13]. A His/His/His triad coordinates Fe(II), to which l-cysteine binds in a bidentate fashion via its amino and thiolate groups, prior to O_2_. Despite significant effort, their catalytic mechanism remains unsolved (possibilities are discussed in [13]), however proposed pathways commonly invoke initial formation of a transient Fe(III)-superoxo intermediate. For mouse CDO this species has been trapped and spectroscopically characterised, albeit having been artificially generated by Fe(II) reduction and superoxide addition [14].

### 2-Hydroxyethylphosphonate dioxygenase

In addition to enzymes that oxidise substrates in which radical intermediates can be stabilised (e.g. aromatic substrates or cysteine), and 2OG/pterin-dependent oxygenases (where Fe(IV)-oxo formation for C—H activation is facilitated by cosubstrate oxidation), some non-heme iron-dependent oxygenases are proposed to directly cleave C—H bonds in aliphatic substrates directly via superoxo intermediates [15]. Although trapping and characterisation of such intermediates remains a challenge, recently reported double kinetic isotope experiments with 2-hydroxyethylphosphonate dioxygenase (HEPD, which catalyses hydroxymethylphosphonate formation in the biosynthesis of the herbicide phosphinothricin, Figure 1f) indicate that substrate activation (requiring cleavage of the HEP C2 C—H bond) must occur prior to the irreversible oxygen reduction step, supporting the use of an Fe(III)-superoxo intermediate to achieve C—H cleavage [16].

Overall, recent studies provide experimental support for some of the lower-valent intermediates proposed to activate substrate by this enzyme family, particularly from *in crystallo* kinetic studies [8•, 9]. Ultimately however, the electronic nature of these reactive iron-oxo intermediates should be confirmed spectroscopically. For Fe(III)-superoxo species this has been achieved in modified enzyme systems, but demonstration of the ‘native’ use of this intermediate remains elusive.

## Non-heme iron oxygenases in plant biology

Here we highlight recent advances in our understanding of selected plant non-heme iron-dependent oxygenases, including those of potential agrochemical/agricultural interest, summarised with further examples in Table 1.

### Carotenoid cleavage dioxygenases

**Figure 2:**
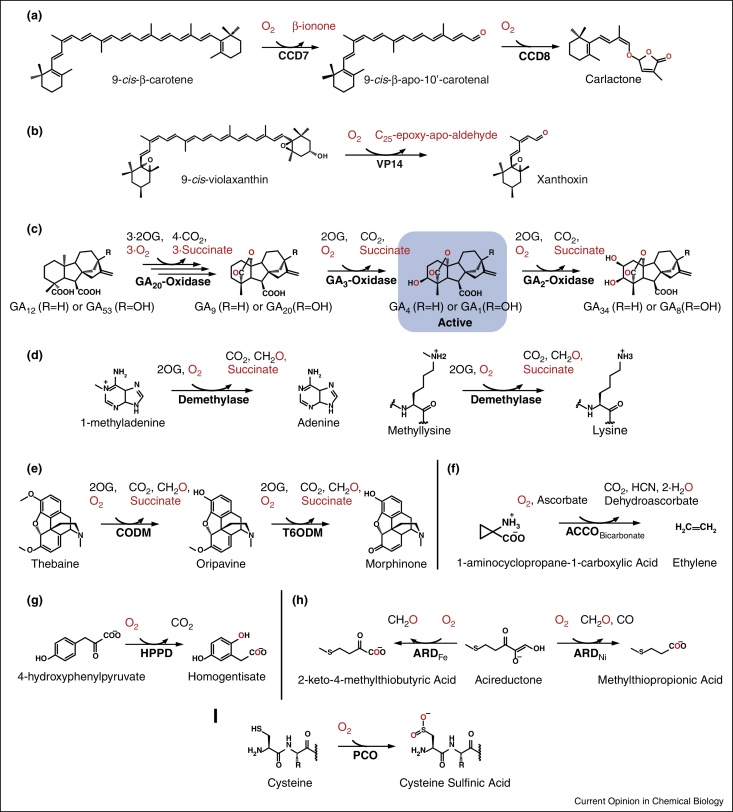
Reactions catalysed by plant non-heme iron-dependent oxygenases, following the route of molecular oxygen (red). **(a)** Carotenoid cleavage dioxygenases 7 and 8 (CCD7 and CCD8 respectively) catalyse the oxidation of 9-*cis*-β-carotene to carlactone. **(b)** The 9′-*Cis*-Epoxycarotenoid Dioxygenase (NCED) VP14 catalyses the oxidative cleavage of 9-*cis*-violaxanthin to xanthoxin. **(c)** Gibberellin (GA) Oxidases catalyse the biosynthesis (GA_20_ and GA_3_-Oxidase) and catabolism (GA_2_-Oxidase) of active GA's (GA_1_ and GA_4_). **(d)** N-demethylases catalyse demethylation of methylated nucleotide bases (left) and histone lysines/arginines (right). **(e)** Codeine O-demethylase (CODM) and thebaine 6-O-demethylase (T6ODM) catalyse the demethylation of thebaine to morphinone. **(f)** 1-aminocyclopropane-1-carboxylic acid oxidase (ACCO) catalyses the breakdown of 1-Aminocyclopropane-1-carboxylic Acid to ethylene. **(g)** 4-hydroxyphenylpyruvate dioxygenase (HPPD) catalyses the oxidation of 4-hydroxyphenylpyruvate to homogentisate. **(h)** Acireductone dioxygenases (ARD) catalyse the production of 2-keto-4-methylthiobutyric acid from acireductone. **(i)** Plant cysteine oxidases (PCO) catalyse the oxidation of N-terminal cysteinyl residues to cys-sulfinic acid.

Carotenoid cleavage dioxygenases (CCDs) catalyse the oxidative cleavage of double bonds in carotenoid backbones to form apocarotenoid precursors for the biosynthesis of plant signalling molecules, including strigolactones and abscissic acid (ABA). Strigolactones, known to act as root-derived signals for symbiotic fungi and parasitic plant germination, have recently been identified as growth hormones that regulate shoot branching [17]. The strigolactone biosynthetic pathway includes 9-*cis*-β-carotene oxidation to 9-*cis*-β-apo-10′-carotenal, catalysed by CCD7, followed by a double oxygenation reaction to form carlactone, catalysed by CCD8 (Figure 2a) [18^••^]. The first committed step in the biosynthesis of ABA, a phytohormone that inhibits growth, is catalysed by 9′-cis-epoxycaroteniod dioxygenases (NCEDs, Figure 2b)[19]. Other CCDs have roles in saffron biosynthesis [20], tuber development in potatoes [21] and formation of isoprenoid volatiles in tomatoes [22].

Selective inhibition of CCDs would be a valuable agrochemical tool to modify plant growth and development characteristics, thus structural and mechanistic studies are important. A crystal structure of the maize NCED Vp14 [23] reveals a seven-bladed β-propeller scaffold, with a helical dome over the upper face. The active site Fe(II) is located in the centre of the propeller, ligated to four histidine residues from separate blades, with H_2_O and O_2_ occupying the remaining coordination sites in the (substrate-free) crystal structure [23]. Three second sphere glutamate residues, one from each of the remaining blades, are also highly conserved, though their role in catalysis is unknown. Oxygen isotope experiments, where solvent (i.e. H_2_^16−^O) back-exchange of cleavage products is accounted for, suggests a dioxygenation reaction mechanism [24, 25•]. However mechanistic understanding of the CCDs lags behind many other non-heme iron-dependent oxygenases (reviewed in [26]). Similarly, inhibitor studies are at an early phase, though mimics of NCED substrates and (potential) intermediates reduce ABA production *in planta* [26], and certain hydroxamic acids are capable of selective inhibition of CCD8 [27].

### 2-Oxoglutarate-dependent oxygenases

The 2OG-dependent oxygenases are involved in a number of primary and secondary metabolic functions in plants, recently reviewed in [28^•^]. These include catalysis of key steps in the biosynthesis of gibberellins (GAs), which have important roles in growth and development. GA C20-oxidase and C3-oxidase catalyse the final step in the production of bioactive forms of the GAs, while GA C2-oxidase catalyses the first step in their catabolism (Figure 2c) [29]. Interestingly the IR8 ‘miracle’ rice variant that was integral to the Green Revolution of the mid 20th century was the result of mutations in the GA C20-oxidase isoform found in leaves and stems, which stunted growth (giving stronger stems) while maintaining fertility, allowing more rice to be produced per unit of agricultural land [30]. These included mutations to conserved residues [31], though their biochemical impact on enzyme function has not been rationalised. A dwarf phenotype linked to naturally occurring mutations in GA C20-oxidase genes has also been observed in other species, for example, Arabidopsis and barley [32, 33]. The significant benefits of exploiting these natural variants suggest that targeted manipulation of GA oxidase activity could also result in improved yields. Indeed, 2OG competitive compounds such as daminozide and prohexadione have been used to inhibit GA oxidases, limiting non-productive crop growth and resulting in stem stabilisation [34]. Such 2OG mimics may, however, inhibit other 2OG oxygenases, resulting in non-specific downstream effects, for example, daminozide is a known human histone demethylase inhibitor [35]. Gibberellin-competitive compounds may therefore be more selective for GA oxidase targeting, for example, *exo*-16,17-dihydro GA_20_-acetate has shown inhibition of GA C3-oxidase [36].

2OG oxygenases are also involved in the epigenetic regulation of gene expression via catalysis of N-demethylation reactions in nucleotides and histones (Figure 2d). Plant 2OG-dependent demethylases are less well-characterised than their mammalian homologues, but there is a growing body of evidence supporting roles for DNA demethylases and particularly for histone-demethylases in processes including flower development and stress tolerance (see [28^•^] and references therein). Interestingly, while histone lysine demethylation is well characterised (especially in humans [37]), a role for histone arginine demethylation in regulating GA signalling and seed germination has been identified in Arabidopsis. On light stimulation, JMJ20 and JMJ22 are activated, removing repressive histone arginine methylation mark and promoting transcription of the genes encoding GA C3-oxidases; GA signalling then promotes seed germination [38]. However, the *in vitro* activity of JMJ20 activity was only demonstrated by western blot analysis and supporting mass spectrometric evidence should be sought to verify this enzyme-mediated arginine demethylation, in order to avoid the controversy that confuses arginine demethylase function in humans [37]. Biochemical/biophysical studies of plant N-demethylases are limited to date (besides a reported structure of the rice H3K4 demethylase JMJ703 catalytic domain [39]) but represent an exciting area for future research. While the complexities involved in selective 2OG-dependent demethylase inhibition may prove challenging, epigenetic regulation by plant N-demethylases will undoubtedly prove to be very important in regulation of gene expression, as for their mammalian counterparts [37].

The first reported O-demethylation reactions catalysed by 2OG oxygenases have recently been identified: In opium poppy plants, thebaine conversion to codeine and morphine is catalysed by thebaine 6-O-demethylase and codeine O-demethylase respectively (Figure 2e) [40^••^]. Formaldehyde detection upon O-demethylation indicated that the mechanism of O-demethylation is homologous to that of 2OG oxygenase-catalysed N-demethylation, that is, methyl hydroxylation followed by formaldehyde elimination [40^••^]. These enzymes, and also 2OG-dependent protopine dealkylase, were subsequently found capable of catalysing a range of dealkylation reactions, including O,O-demethylenation, in the biosynthesis of multiple benzylisoquinoline alkaloids in opium poppy [41]. Further, a 2OG-dependent flavone 7-O-demethylase has been identified with a role in flavonoid biosynthesis in sweet basil [42]. The identification of these O-demethylating enzymes adds to the catalytic repertoire of the already diverse 2OG oxygenases, while their potential for manipulation, for example, to inhibit morphine biosynthesis, has important societal and pharmaceutical implications [40••, 43].

### 2-Oxoglutarate dependent ‘like’ oxygenases

1-Aminocyclopropane-1-carboxylic acid oxidase (ACCO) catalyses the final step in biosynthesis of the signalling hormone ethylene (Figure 2f), which influences multiple processes including stress responses and fruit ripening. Although structurally related to the 2OG oxygenases [44], ACCO does not follow the consensus reaction mechanism whereby Fe(II)-coordinated 2OG acts as the two-electron donor to enable O—O scission and formation of a reactive Fe(IV)-oxo species. Instead, ACC binds directly to the Fe(II) via its carboxylate and amino groups, with a closely bound ascorbate acting as an electron donor promoting formation of a reactive Fe intermediate (an Fe(IV)-oxo species is proposed based on kinetic studies [45]) which triggers ACC ring-opening, ethylene and cyanoformate formation. Cyanoformate is proposed to be shuttled away from the enzyme before it decomposes to cyanide and carbon dioxide in the cell medium [46]. Bicarbonate/CO_2_ is required to activate the enzyme, possibly by stabilising the enzyme-substrate complex [47]. Control of ethylene biosynthesis is a major target for regulating fruit ripening, but to date approaches have been predominantly genetic with limited pursuit of small molecule ACCO inhibitors [34].

4-Hydroxyphenylpyruvate dioxygenase (HPPD), present in all living organisms, catalyses the first committed step in l-tyrosine catabolism, converting 4-hydroxyphenylpyruvate (HPP) to homogentisate (Figure 2g). Homogentisate is vital to plant life, as the precursor to tocopherols (plant hormones and antioxidants) and plastiquinone (photosynthetic electron transport and carotenoid biosynthesis). Carotenoid-deficient plants are susceptible to UV damage, bleaching and death, making HPPD an herbicidal target. It represents another ‘2OG-oxygenase-like’ enzyme in that instead of using an α-keto acid from a 2OG co-substrate as the electron donor to cleave O—O, HPPD uses an α-keto acid in the pyruvate substituent of the HPP substrate, coordinated directly to the Fe(II). Nevertheless, the proposed mechanism is similar to the 2OG oxygenases: oxidative decarboxylation of HPP yields 4-hydroxyphenylacetate and CO_2_, with O—O cleavage forming a (predicted) Fe(IV)-oxo species [48] which enables homogentisate formation (detailed mechanistic possibilities are discussed in [49]). The overall structure of HPPD has a vicinal oxygen chelate fold (like some of the extradiol dioxygenases) supporting the active site [49, 50]. Compounds which chelate to the active site Fe(II) have been developed and successfully deployed as HPPD inhibitors, for example, the triketones mesotrione and sulcotrione [49]. The emergence of herbicide resistant weeds, including those resistant to HPPD inhibitors [51], is fuelling research into novel inhibitors, safeners and inhibitor-resistant GM crops [52, 53•].

### Acireductone dioxygenases

Acireductone dioxygenases (ARDs) constitute part of the methionine salvage pathway, which is important in plants in maintaining *S*-adenosylmethionine levels for ethylene production [54]. Interestingly, the active site metal (coordinated by three histidines and a glutamate [55]) can be either Fe(II) or Ni(II), and the nature of the metal determines the catalysed reaction. ARD-Fe(II) catalyses conversion to formate and 2-keto-4-methylthiobutyric acid, the precursor of methionine. ARD-Ni(II) catalyses conversion to CO, formate and methylthiopropionic acid (Figure 2h), which then exits the pathway. The mechanism for the different reactions has been proposed to be due to different modes of substrate ligation resulting in substrate attack at different positions by oxygen, and more recently to hydration of a vicinal triketone intermediate in the ARD-Fe(II) but not the ARD-Ni(II) enzyme (discussed in [56]). This work has been conducted with enzymes from bacteria but mechanistic investigations warrant further pursuit in plants, as ARD and the methionine salvage pathway has a role in abiotic and biotic stress tolerance [57]. Activity has been demonstrated *in vitro* for a rice ARD-Fe(II) [54], but there is no evidence to date for a role of the ARD-Ni(II) form in plants. Nevertheless, the possibility exists that ARD activity, methionine cycling and thus ethylene-mediated stress tolerance could be affected by relative Fe(II) and Ni(II) availability.

### Plant cysteine oxidases

Recent work investigating the hypoxic responses of Arabidopsis thaliana has led to the identification of putative non-heme iron-dependent oxygenases with the capacity to regulate hypoxia-responsive transcription factor levels in an O_2_-dependent manner [58^••^]. These Plant Cysteine Oxidases (PCOs), which have sequence homology with the CDOs [13], catalyse the O_2_-dependent oxidation of N-terminal cysteine residues of Group VII Ethylene Response Factors (Figure 2j, [58^••^] and MD White *et al.*, unpublished), targeting them for proteasomal degradation via the N-end rule pathway [59]. The requirement of the PCOs for O_2_ connects their activity with the plant hypoxic response and suggests they could act as plant O_2_-sensors. Notably, these enzymes are not 2OG-dependent, yet their role in the plant hypoxic response appears to be functionally homologous to the highly O_2_-sensitive 2OG-dependent HIF hydroxylases which regulate the hypoxic response in animals [60, 61]. Biochemical and kinetic characterisation of the PCOs will therefore be intriguing, establishing if they are true O_2_-sensors and thus a possible intervention point for increasing crop tolerance to hypoxic conditions, for example, flooding.

## Summary and future perspectives

Non-heme iron-dependent oxygenases are enzymes of extraordinary catalytic capability and considerable efforts in the field continue to elucidate the diverse strategies they use to activate substrate and achieve oxidation. These enzymes are also fascinating due to the vast array of important biological functions they perform. Structural/functional studies can facilitate manipulation of their activity, either to probe their biological function or to promote a desirable phenotypic effect in the native organism, including for therapeutic purposes. The *in vivo* scope for genetically modifying human/animal enzymatic properties is (currently) limited, thus small molecule/peptidic therapies remain primary targets. In plants however, the possibility exists to both chemically *and* genetically alter enzymatic behaviour. Nature has already demonstrated that modified plant oxygenases can have significant beneficial effects on crop yield [30]. By furthering our understanding of these important enzymes it may therefore be possible to efficiently design targeted variant plants capable of improved yield, stress tolerance or nutrient production, helping to address some of the global challenges of our time.

## References and recommended reading

Papers of particular interest, published within the period of review, have been highlighted as:

• of special interest
•• of outstanding interest

## Figures and Tables

**Table 1:** A summary of the roles of different plant non-heme iron-dependent oxygenases in primary and secondary metabolism and the potential agricultural benefit of their manipulation

Enzyme(s)	Oxygenase class	Biological function	Potential agricultural application(s)	Reference(s)
Flavanone 3-β-hydroxylase (FHT), flavonol synthase (FLS) and anthocyanidin synthase (ANS)	2OG-dependent dioxygenase	Flavonoid (e.g. anthocyanin) biosynthesis	Control of photoinhibition properties, visual characteristics (e.g. colour) and (possibly) antioxidant content	[62]
Feruloyl CoA hydroxylase (F6′H1)	2OG-dependent dioxygenase	Iron deficiency response signalling	Manipulation of environmental compatibility	[63]
9′-Cis-epoxycaroteniod dioxygenase (NCED)	Carotenoid cleavage dioxygenase	Abscissic acid biosynthesis	Control of abiotic stress tolerance (e.g. drought) and growth and development characteristics	[19, 23, 24]
1-Aminocyclopropane-1-carboxylic acid oxidase (ACCO)	2OG-dependent ‘like’ dioxygenase	Ethylene biosynthesis	Manipulation of fruit ripening, abiotic stress tolerance (e.g. flooding) and senescence.	[44, 45]
9-Lipoxygenase (9LOX) and 13-lipoxygenase (13LOX)	Lipoxygenase	Oxylipin (e.g. jasmonic acid) and volatile hydrocarbon biosynthesis	Control of herbivore/microbial defence properties, flavour intensity and abiotic stress tolerance (e.g. high salinity)	[64]
Gibberellin C2 (GA_2_), _C3_ (GA_3_) and C20 (GA_20_) oxidase	2OG-dependent dioxygenase	Gibberellin biosynthesis (GA_3_ and GA_20_ oxidase) and catabolism (GA_2_ oxidase)	Manipulation of growth and development characteristics	[29, 30, 33]
Salicylic acid 3-hydroxylase (S3H)	2OG-dependent dioxygenase	Salicylic acid catabolism	Control of biotic/abiotic stress tolerance (e.g. temperature) and senescence	[65]
Carotenoid cleavage dioxygenase 7 (CCD7) and 8 (CCD8)	Carotenoid cleavage dioxygenase	Strigolactone (e.g. orobanchol) biosynthesis	Manipulation of parasitic plant interaction, symbiotic fungal relationships and growth and development characteristics (e.g. shoot and root branching)	[17, 18••]
Dioxygenase for auxin oxidation (DAO)	2OG-dependent dioxygenase	Auxin (e.g. indole-3-acetic acid) catabolism	Control of fruit development and tropic responses (e.g. hydrotropism)	[66]
Plant cysteine oxidase 1-5 (PCO1-5)	Cysteine dioxygenase	Hypoxic response regulation (oxygen sensing?)	Manipulation of abiotic stress tolerance (hypoxia)	[58••, 59]
Acireductone dioxygenase (ARD)		Methionine salvage/ethylene production	Manipulation of fruit ripening, abiotic stress tolerance (e.g. flooding) and senescence.	[57]
4-Hydroxyphenylpyruvate dioxygenase (HPPD)	2OG-dependent ‘like’ dioxygenase	Tyrosine catabolism/tocopherol and plastiquinone production	Control of antioxidant/nutritional content and photoinhibition properties. Herbicide target.	[52, 53•]
3,4-Dihydroxyphenylalanine dioxygenase (DOD)	Catechol dioxygenase (extradiol)	Betalain (e.g. betanin) biosynthesis	Manipulation of photoinhibition properties and visual characteristics (e.g. colour)	[67]
Histone arginine demethylases JMJ20 and JMJ22	2OG-dependent demethylase	Epigenetic regulation (e.g. gibberellin signalling)	Control of growth and development characteristics (e.g. seed germination)	[28^•^]
